# Effects of high-intensity interval versus mild-intensity endurance training on metabolic phenotype and corticosterone response in rats fed a high-fat or control diet

**DOI:** 10.1371/journal.pone.0181684

**Published:** 2017-07-20

**Authors:** Youqing Shen, Guoyuan Huang, Bryan P. McCormick, Tao Song, Xiangfeng Xu

**Affiliations:** 1 School of Physical Education, Hubei University of Education, Wuhan, Hubei Province, China; 2 Pott College of Science, Engineering, & Education, University of Southern Indiana, Evansville, Indiana, United States of America; 3 School of Public Health, Indiana University-Bloomington, Bloomington, Indiana, United States of America; 4 School of Physical Education, Jianghan University, Wuhan, Hubei Province, China; 5 School of Physical Education, Wuhan Sports University, Wuhan, Hubei Province, China; Universidad Pablo de Olavide, SPAIN

## Abstract

The aim of the present study was to compare the effects of high-intensity interval training (HI) to mild-intensity endurance training (ME), combined with a high-fat diet (HFD) or control diet (CD) on metabolic phenotype and corticosterone levels in rats. Fifty-three rats were randomized to 6 groups according to diet and training regimen as follows: CD and sedentary (CS, *n* = 11), CD and ME (CME, *n* = 8), CD and HI (CHI, *n* = 8), HFD and sedentary (HS, *n* = 10), HFD and ME (HME, *n* = 8), and HFD and HI (HHI, *n* = 8). All exercise groups were trained for 10 weeks and had matched running distances. Dietary intake, body composition, blood metabolites, and corticosterone levels were measured. Histological lipid droplets were observed in the livers. The HFD led to hyperglycemia, hyperlipidemia and higher body fat (all, *P* < 0.01, η^2^ > 0.06), as well as higher corticosterone levels (*P* < 0.01, η^2^ = 0.09) compared with the CD groups. Exercise training improved fat weight, glucose, and lipid profiles, and reduced corticosterone levels (*P* < 0.01, η^2^ = 0.123). Furthermore, body and fat weight, serum glucose and triglycerides, lipid content in the liver, and corticosterone levels (*P* < 0.05) were lower with HI training compared to ME training. Reductions in HFD-induced body weight gain, blood glucose and lipid profiles, and corticosterone levels, as well as improvements in QUICKI were better with HHI compared to HME. Correlation analyses revealed that corticosterone levels were significantly associated with phenotype variables (*P* < 0.01). Corticosterone level was inversely correlated with QUICKI (*r* = −0.38, *P* < 0.01). Altogether, these results indicate that HFD may elicit an exacerbated basal serum corticosterone level and thus producing a metabolic imbalance. Compared with ME training, HI training contributes to greater improvements in metabolic and corticosterone responses, leading to a greater reduction in susceptibility to HFD-induced disorders.

## Introduction

The prevalence of high-fat diets (HFD) is a major risk factor contributing to an obesity epidemic and associated homeostatic imbalance, globally. Feeding behavior can induce metabolic and neuroendocrine responses, producing divergent changes to metabolic profiles and hormone secretions [1, 2]. Animals and human beings who consume HFD are more likely to develop obesity, insulin resistance, and type 2 diabetes mellitus (T2DM). A high-fat diet is commonly used in rodent research to examine susceptibility to metabolic disorders and associated chronic diseases, as characterized by abnormal body composition, blood glucose and lipid profiles, and excessive lipid accumulation in the adipose and hepatic tissues [3, 4]. Moreover, consuming excessive amounts of food with saturated fat may affect homeostasis of the whole body, including the hypothalamic-pituitary-adrenal (HPA) axis [5, 6]. Corticosterone in rodents (cortisol in humans) is a glucocorticoid (GC), which is secreted by the adrenal glands and affects gluconeogenesis and fatty acid release, and is the end product of HPA axis. Corticosterone modulates metabolism and biological functions and responds differently depending on internal and external environmental changes [7].

A physiological level of corticosterone is required to maintain metabolic activity and homeostasis, whereas aberrant secretion is linked to the development of obesity and comorbidities [6, 8, 9]. Previous studies have reported the pathophysiological overlap of the development of obesity and associated metabolic diseases to the HPA axis [10, 11]. A possible mechanism for HFD-induced obedity is the activation of the stress axis, which is essential for affecting metabolism and hormone release leading to an increase in the susceptibility for development of metabolic diseases [12, 13]. Because of neuroendocrine disruptions in the biological response to stress, HFD increases circulating corticosterone, which induces feedback in the hyperactivity of the HPA axis [13, 14]. Miller and O’Callaghan (2002) reported that an over- or underproduction of cortisol in humans resulted in Cushing’s syndrome or Addison’s disease and that even less severe dysregulation of the HPA axis could have adverse health consequences associated with abdominal adiposity and additional metabolic complications [15]. The influence of a high-fat diet on the basal activity of the HPA axis and corticosterone secretion in rodents, as well as the underlying mechanisms of the HFD remains inconclusive. For example, some studies have shown an increase in circulating corticosterone [13, 16], whereas others have found a decrease or no significant influence on circulating corticosterone in rodents fed a high-fat diet [4, 17, 18].

Current evidence suggests that exercise training may reduce susceptibility to metabolic disorders and enhance habituation to repeated stress [19–21]. Mild- to moderate-intensity endurance training (ME) was previously considered effective for attenuating obesity, hyperinsulinemia, and lipid accumulation in peripheral organs [22, 23]. Recent studies have indicated that high-intensity interval training (HI) can induce similar or better benefits compared with other types of exercise, while promoting exercise participation and improving health by decreasing fat mass, enhancing insulin sensitivity, and influencing muscle metabolic adaptations[24, 25]. Current evidence suggests that voluntary wheel running has an impact on stress-induced metabolic disorders, attenuates obesity, and improves the metabolic profiles associated with HFD and GC-treated rats [1, 26]. However, adequate exercise may play an important role in alleviating stress-related behavior abnormalities and improving the functional state of the HPA axis [27, 28]. Together, this mixed and controversial data suggest that there is a potential alteration of stress-related physiological responses resulting from exercise and HFD feeding. Although exercise is linked to metabolic health benefits, it is surprising that few studies have explored interactions between different exercise regimens with or without a high-fat diet and corticosterone responses, either independently or interactively. For example, it remains unclear whether different exercise regimens lead to similar metabolic and stress responses and adaptations in the presence of a high-fat diet.

The present study was conducted to determine the influence of ME and HI training combined with a high-fat diet or CD on metabolic and corticosterone responses. We hypothesized that (1) diet and exercise, as main and interactive factors, would have major effects on metabolic phenotype and corticosterone release. (2) A high-fat diet would induce an adverse metabolic phenotype and high-level of corticosterone secretion. (3) HI exercise would have a greater influence on metabolic and corticosterone responses and adaptation, possibly due to the overlap of metabolic and neuroendocrine effects. This study was also designed to determine the correlation between circulating corticosterone and metabolic variables, including fat weight, serum glucose, QUICKI and triglycerides, which may reflect an inherent relationship between metabolic activities and neuroendocrine responses.

## Materials and methods

### 2.1. Ethics statement

Ethical approval for this study was obtained from the Institutional Animal Care and Use Committee of Jianghan University, Hubei Province, China (Permit Number: SCXK20080004). All experimental procedures and facilities were operated in accordance with the U.S. National Institutes of Health Guide for the Care and Use of Laboratory Animals.

### 2.2. Animals and housing conditions breeding

Fifty-three male Sprague-Dawley rats (8–10 weeks of age, weighing 190 ± 15 g) were supplied by a local breeding facility (Wuhan University Center for Animal Experiment/A3-Lab, Wuhan, Hubei Province, China). Rats were housed three to four per cage under standard conditions: constant temperature (23 ± 1°C) and humidity (40–60%), *ad libitum* access to food and water, and 12 h light–dark cycle (lights on 19:00–07:00). Upon arrival, rats were acclimated to environment and circadian rhythm for 1 week prior to intervention. Rats were randomly assigned into six groups according to diet and exercise protocol, as follows. Control diet and sedentary (CS, *n* = 11), control diet with mild-intensity endurance training (CME, *n* = 8), control diet with high-intensity interval training (CHI, *n* = 8), high-fat diet and sedentary (HS, *n* = 10), high-fat diet with mild-intensity endurance training (HME, *n* = 8), and high-fat-diet with high-intensity interval training (HHI, *n* = 8). Rats were pair-fed with either a high-fat diet (D12451; 4.73 kcal/g, energy content: 45% fat, including 12.3% from soya oil and 87.75% saturated fat from lard; 35% carbohydrates, including 21.1% from corn starch, 28.9% from Maltodextrin 10, and 50% from sucrose; and 20% protein) or CD (D12450B; 3.85 kcal/g, energy content: 10% fat, including 55.6% from soya oil and 44.4% saturated fat from lard; 70% carbohydrates, including 45% from corn starch, 5.0% from Maltodextrin 10, and 50% from sucrose; and 20% protein) (Research Diets, Inc. New Brunswick, NJ, United States). These matched and purified ingredient-based diets are the standard for research in the field of obesity, T2DM, and metabolic syndrome. Rats were weighed twice per week and their food was changed twice daily. Daily food intake was measured by weighing total amount of food provided to the rats and subtracting the remaining food in the cage every 24 hours. Cage bedding was searched manually for leftover pieces of pellets to take into account any food spillage and hoarding.

### 2.3. Treadmill training protocols

Training was performed on a specialized treadmill that had a motor-driven grade and time setting (Zhenghua Biological Equipment Company, Anhui, China). The treadmill consisted of eight parallel runways (each running track = 100 cm × 9.6 cm × 12 cm) with two transparent outer covers (50 cm × 88 cm), which prevented the rats from falling off the treadmill. Animals in the exercise groups were acclimated to the treadmill for 15 min/d at a speed of 5 m/min for 1 week before starting the exercise program. In accordance with nocturnal habits, all rats were trained during the dark cycle (07:00–11:00 AM). Rats attempting to rest were encouraged to continue running by gently tapping the feet with a bristle brush on the rear grid. After acclimation and 2 days of rest, rats in the exercise groups underwent training 5 days/week for 10 weeks with matched running distances. Exercise intensity was performed and adjusted, as described previously [29]. Animals were placed on their respective dietary and training regimens for 10 weeks. The running speed for ME training started at 10 m/min (5-degree inclination), was increased by 2 m/min per week over the first 4 weeks, and then maintained at 16 m/min for the remaining 6 weeks. Duration of the constant, mild intensity of ME training was of 40 min/day. The HI groups trained in interval sessions consisting of successive 30 s periods of heavy intensity interspersed with 10 s of sedentary recovery. Each session began at a speed of 20 m/min (5-degree inclination), was increased by 4 m/min per week over the first 4 weeks, and maintained at 32 m/min for the remaining 6 weeks. Duration of HI training was of 20 min/day. To account for the stress induced by animal handling, sedentary groups also were placed in a stationary treadmill for acclimation [30].

### 2.4. Blood sampling and serum analyses

After 10 weeks, animals were fasted overnight, and then anesthetized with pentobarbital sodium (40 mg/kg, i.p.). After the animals were completely anesthetized, the abdominal fur was shaved off. An abdominal midline incision was performed and the abdominal cavity was opened. Blood was rapidly drawn from the abdominal aorta using 10ml vacuum blood collection tube. All samples were collected between 08:00 am and 10:00 am to avoid temporal influences on blood metabolites. Serum was collected after samples were centrifuged at 3000 rpm at 4°C for 15 minutes. Serum samples were collected and fasting glucose, triglycerides (TG), total cholesterol (TC), high-density lipoprotein cholesterol (HDL-C), and low-density lipoprotein cholesterol (LDL-C) were measured using an automated analyzer (AEROSET Automatic Biochemical Analyzer, Abbott Company, IL, USA). Commercially rat-specific enzyme-linked immunosorbent assay (ELISA) kits were used to determine serum insulin (Cat. No. EZRMI-13K, Linco Research, St. Charles, MO, USA) and corticosterone level (Cat. No. E-2724, Shanghai Meilian Bio-tech Company, China). The quantitative insulin sensitivity check index (QUICKI) was calculated according to the formula, as described previously QUICKI = 1/ [log (*I0*) + log (*G0*)], where *I0* is fasting insulin (IU/ml) and *G0* is fasting glucose (mg/dl) [31].

### 2.5. Tissue collection and histological analysis

Livers and fat pads in the mesenteric (MES), retroperitoneal (RET), and epididymal (EPI) regions were collected and weighed. Liver samples were fixed in 10% neutral buffered formaldehyde, embedded in paraffin, and sectioned at a thickness of 4 μm on a microtome (Leica RM 2016, Leica Biosystems, Wetzlar, GER) for standard hematoxylin-eosin (H&E) staining. Histological samples were imaged using an electron microscope (Olympus DP72, Olympus Corporation, Tokyo, Japan).

### 2.6. Statistical analysis

Statistical analyses were performed using PASW Statistics for Windows Version 18.0. Descriptive statistics were presented as mean ± SD, with uncertainty in the estimates expressed as 95% confidence intervals. Repeated measures analysis of variance (ANOVA) was used to assess weekly body weight curves. Greenhouse-Geisser corrections were used when Mauchly's test of sphericity was violated. Significant differences were followed by post-hoc LSD tests. A two-way factorial ANOVA with multiple comparisons and post hoc Scheffé’s tests were used to test the main effect of diet and/or exercise and the interaction effect on the outcome variables. Eta Squared (η^2^) was used to estimate effect size. The following values were defined to interpret the strength of the η^2^ effect: small (0.01), medium (0.06), and large (0.14) [32]. Separate one-way ANOVA with post hoc Scheffé’s test was conducted to identify the differences among treatment groups following the same diets. Pearson-product moment correlation coefficients were computed to examine the relationships between metabolic parameters and circulating corticosterone. A value of *P* < 0.05 was considered statistically significant.

## Results

### 3.1. Food consumption and caloric intake

HFD group rats ate significantly less food than rats in the CD groups (Table 1). However, caloric intake was greater in the HFD groups than in the CD groups. Exercise or interactions had no significant main effect on food and caloric intake. The mean differences in total caloric intakes were not statistically significant when comparing the CD groups to each other or the HFD groups to each other.

**Table 1:** Characteristics of dietary consumption, body weight, and the relative variables induced by different diets and exercises.

	CS	CME	CHI	HS	HME	HHI	Diet		Exercise		Diet × exercise	
	n = 11	n = 8	n = 8	n = 10	n = 8	n = 8	Main effect	η^2^	Main effect	η^2^	Interaction	η^2^
**Food intake (g/d)**	22.55±1.80	22.32±1.93	21.92±2.10	19.30±1.90	19.52±2.06	19.01±2.29	F = 32.77, P<0.01	0.374	F = 0.34, P>0.05	0.007	F = 0.07, P<0.05	0.002
**Caloric intake (kcal/d)**	86.81±6.92	85.92±7.42	84.41±8.09	91.68±9.04	92.72±9.79	90.30±10.87	F = 6.66, P<0.05	0.108	F = 0.32, P>0.05	0.010	F = 0.06, P<0.05	0.002
**Initial weight (g)**	205.45±24.45	208.63±11.84	204.38±11.80	203.10±12.07	208.75±6.76	203.25±2.66						
**Final weight (g)**	425.95±29.95	417.94±31.57	417.31±18.63	493.80±58.91	468.75±47.68	406.63±53.65**,^##^	F = 9.25, P<0.01	0.120	F = 5.78, P<0.01	0.150	F = 4.06, P<0.05	0.105
**Weight gain (Δ) (g)**	220.50±37.93	209.31±25.92	212.94±20.42	290.70±50.61	260.00±43.20	203.38±52.63**	F = 10.88, P<0.01	0.135	F = 6.19, P<0.01	0.154	F = 4.57, P<0.05	0.113

*Note*: CS = control diet and sedentary; CME = control diet with ME exercise; CHI = control diet with HI exercise; HS = high-fat diet and sedentary; HME = high-fat diet with ME exercise; HHI = high-fat diet with HI exercise; n = number; Δ = weight gain; Value = mean ± SD; η^2^ indicates effect size. Statistical significance using one-way ANOVA: * vs the HS group (**, *P* < 0.01); # vs the ME group (## *P* < 0.01).

### 3.2. Body weight and weight changes

**Fig 1:**
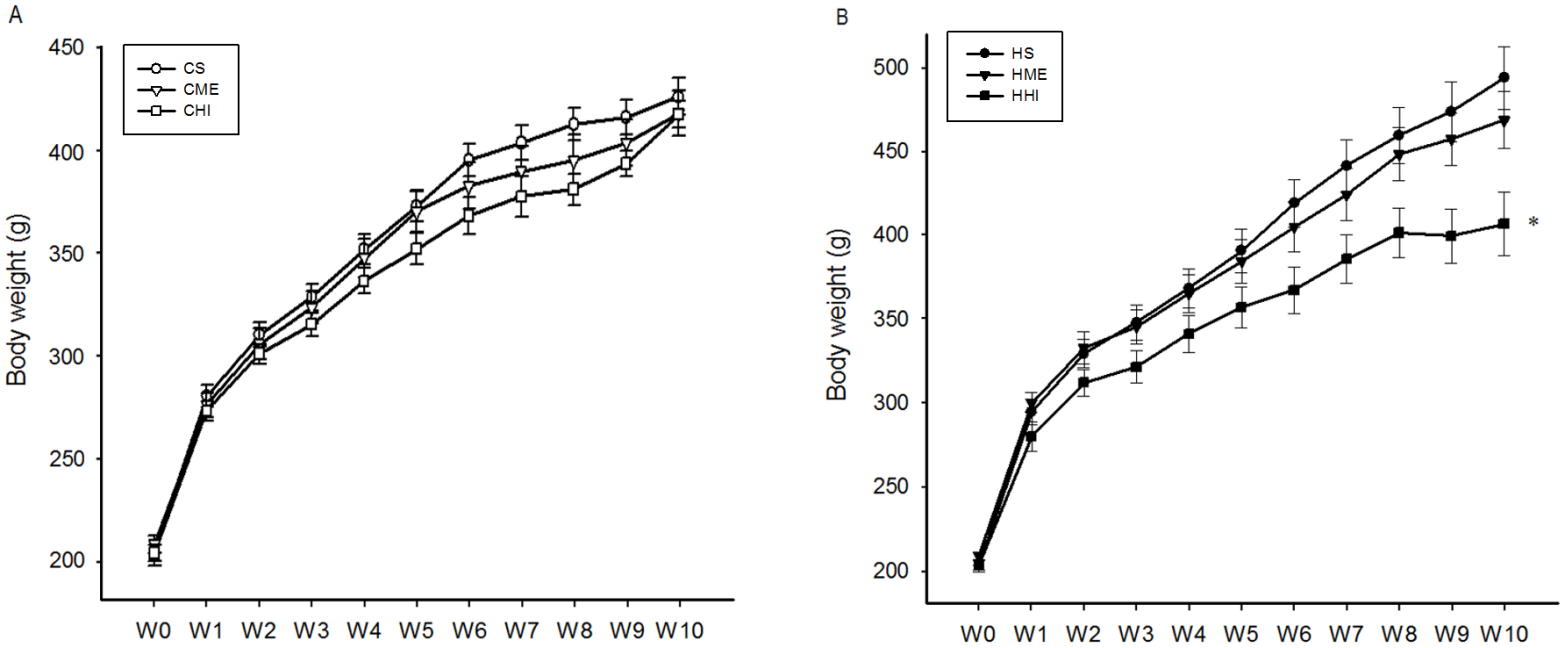
Body weight changes in the CD group and the HFD group during the 10-week intervention period. A. Body weight increased in the CD groups during the 10-week intervention, however there was no mean difference among the CD groups. B. Body weight increased progressively in the HFD groups, though the magnitudes and rates were considerably different among the three groups, with a significant difference in weight gain observed in the HS compared with the HHI group. CS = control diet and sedentary; CME = control diet with ME exercise; CHI = control diet with HI exercise; HS = high-fat diet and sedentary; HME = high-fat diet with ME exercise; HHI = high-fat diet with HI exercise. Statistical significance using repeated measures ANOVA: * vs sedentary group (*, *P* < 0.05).

All groups were similar in initial body mass and had increasing trends in body weight gains (Table 1 and Fig 1). However, the magnitudes of the weight increases were considerably inconsistent among different groups. The order from lowest to highest was CHI, CME, and CS in CD groups, and HHI, HME, and HS in HFD groups. Analysis with repeated measures ANOVA revealed a significant difference between the HS and HHI group (P < 0.05). The significant main effects of diet, exercise and interactions (diet × exercise) were observed in the final weight and weight gain (Δ) (Table 1). Notably, HI training had more obvious effects on reduction of body weight and weight gain compared with ME training (HI vs sedentary, *P* < 0.01, CI: [−82.11 to −10.47]; ME vs sedentary, *P* > 0.05, CI: [−50.74 to 20.90]).

The final weight in the HS group was more than 15.9% higher than in the CS group. Significant mean differences in final weight (F = 5.95, *P* < 0.01) and weight gain (F = 7.10, *P* < 0.01), compared to the baseline values, were found in the HFD groups. However, there were no statistically significant differences in final weight and weight gain vs. baseline in the CD groups. The HHI group had a significantly lower final weight (*P* < 0.01, CI: [−154.30 to −20.05]) and weight gain (*P* < 0.01, CI: [−148.29 to −26.36]) compared with the HS group.

### 3.3. Fat weights

**Fig 2:**
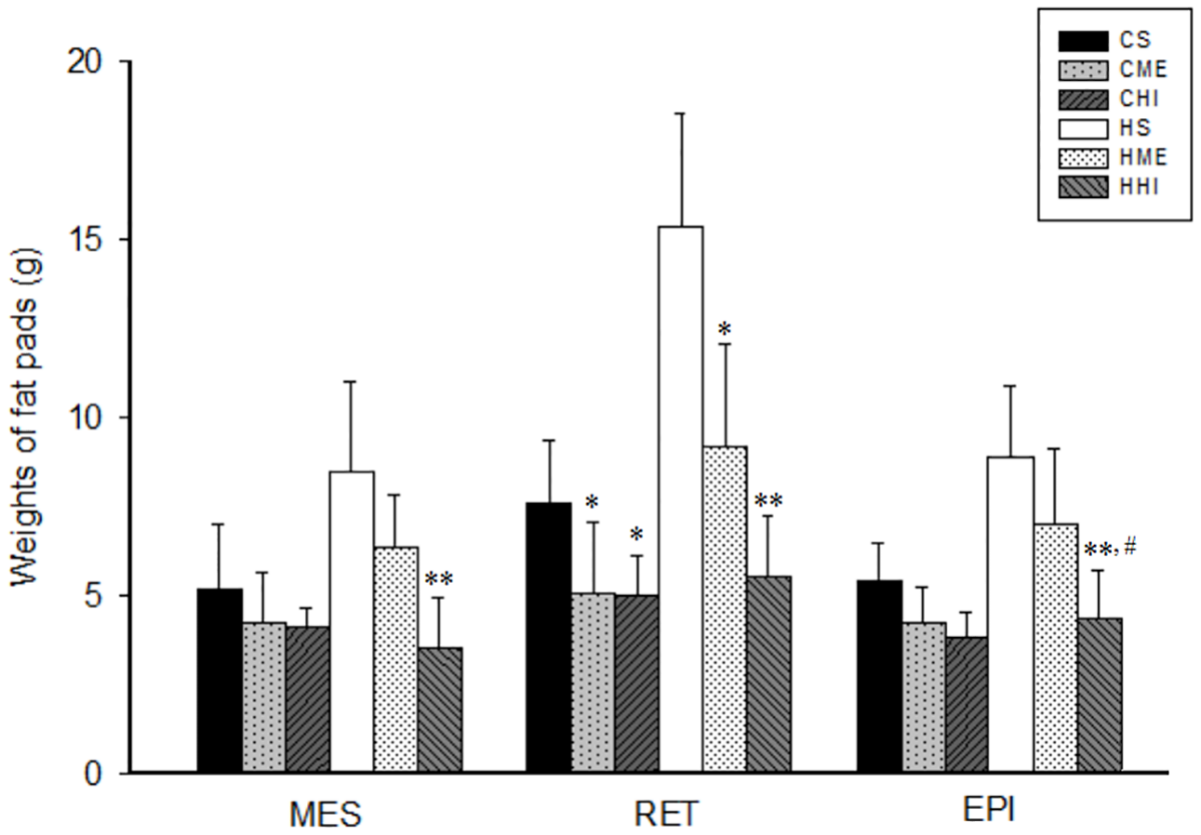
Comparisons of fat pad weights in different regions. RET weight was lower in CME and CHI rats compared to CS rats (*P* < 0.05). Fat pad weights in the MES, RET, and EPI regions were lower in the HHI group compared to the HS group (*P* < 0.01). RET fat weight was lower in the HME group than in the HS group (*P* < 0.05) and EPI fat weight was higher in the HME than in the HHI group. MES, mesenteric region; RET, retroperitoneal region; EPI, epididymal region. CS = control diet and sedentary; CME = control diet with ME exercise; CHI = control diet with HI exercise; HS = high-fat diet and sedentary; HME = high-fat diet with ME exercise; HHI = high-fat diet with HI exercise. Statistical significance using one-way ANOVA: * vs sedentary group (*, *P* < 0.05; **, *P* < 0.01); # vs the ME group (#, *P* < 0.05).

Diet and exercise, independently and interactively, significantly affected fat weight (Table 2). Body fat, as measured by visceral fat pad weight, was increased by the HFD and reduced by exercise. Analysis with one-way ANOVA revealed a significant effect of exercise on fat weight in the CD (F = 5.61, *P* = 0.01) and the HFD groups (F = 18.26, *P* < 0.01). Comparison analyses revealed significant differences among trained rats (HI vs sedentary, *P* < 0.01, CI: [−16.58 to −7.40]; HI vs ME, *P* < 0.05, CI: [−9.85 to −0.07]; ME vs sedentary, P < 0.01, CI: [−11.62 to −2.45]). Both the HHI (*P* < 0.01, CI: [−27.75 to −10.93]) and HME group (*P* < 0.01, CI: [−18.55 to −1.73]) had significantly less fat weight compared to the HS group. Furthermore, the HHI group had a significantly lower fat weight than the HME group (*P* < 0.05, CI: [−18.06 to −0.34]). Similarly, the CHI (*P* < 0.05, CI: [−9.96 to −0.71]) and CME (*P* = 0.05, CI: [−9.24 to −0.01]) groups had lower fat weights than the CS group. Compared with HS rats, HHI rats had lower fat pad weights in the MES, RET, and EPI regions (*P* < 0.01), while HME was effective at reducing RET fat weights (Fig 2). The HHI group rats had lower EPI pad weights than HME group rats (*P* < 0.05). Both HI and ME training reduced the RET pad weights in the CD groups (*P* < 0.05).

**Table 2:** Characteristics and comparisons of fat weight and liver weight.

	CS	CME	CHI	HS	HME	HHI	Diet		Exercise		Diet × exercise	
	n = 11	n = 8	n = 8	n = 10	n = 8	n = 8	Main effect	η^2^	Main effect	η^2^	Interaction	η^2^
**Fat weight (g)**	17.90±4.35	13.29±4.25*	12.57±2.18*	32.47±7.43	22.33±7.14*	13.13±5.36**,^#^	F = 28.31, P<0.01	0.202	F = 23.88, P<0.01	0.341	F = 7.46, P<0.01	0.106
**Liver weight (g)**	10.42±1.20	10.43±1.50	10.32±0.67	12.94±1.72	11.84±1.76	9.99±1.13**	F = 9.70, P<0.01	0.123	F = 5.48, P<0.01	0.139	F = 4.79, P<0.05	0.122

*Note*: CS = control diet and sedentary; CME = control diet with ME exercise; CHI = control diet with HI exercise; HS = high-fat diet and sedentary; HME = high-fat diet with ME exercise; HHI = high-fat diet with HI exercise; n = number; Value = mean ± SD; η^2^ indicates effect size. Statistical significance using one-way ANOVA: * vs sedentary group (*, *P* < 0.05; **, *P* < 0.01); # vs the ME group (#, *P* < 0.05).

### 3.4. Liver weight and lipid accumulation

Liver mass was significantly higher in HFD rats compared with CD rats (Table 2). HI training had pronounced effect on liver weight when liver weight was compared between exercise and sedentary groups (HI vs sedentary, *P* < 0.01, CI: [−2.23 to −0.26]; ME vs sedentary, *P* > 0.05, CI: [−1.65 to 0.69]). Liver weight in the HHI group was significantly lower than in the HS group (*P* < 0.01, CI: [−4.91 to −0.99]).

**Fig 3:**
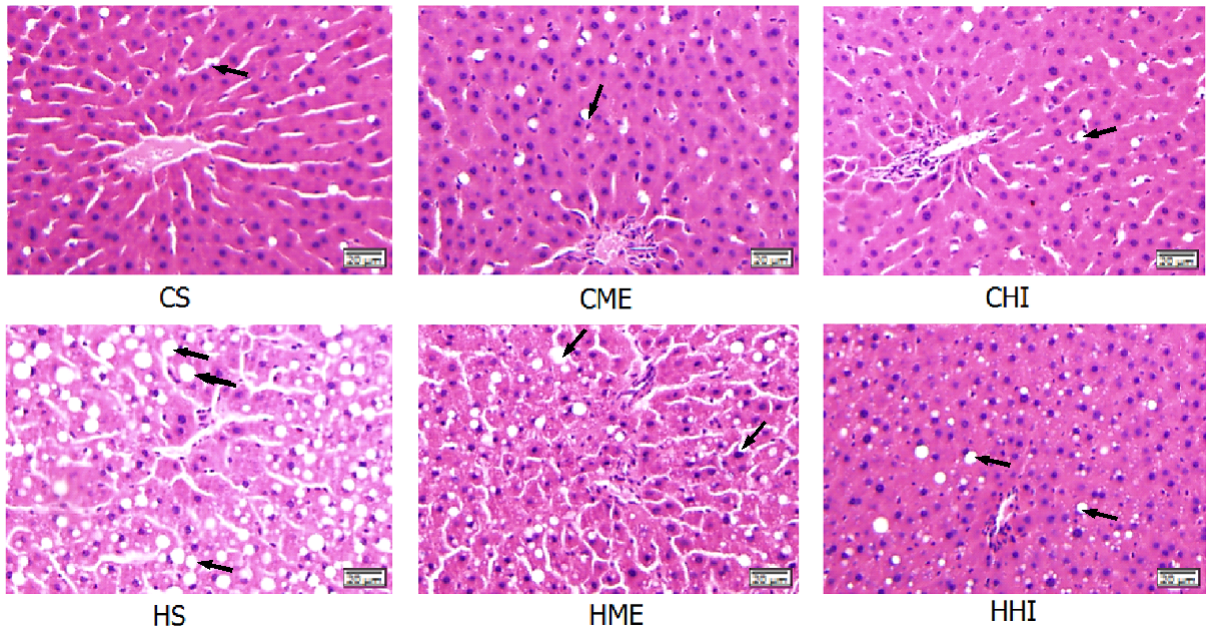
Histological changes in livers of rats in the six groups after the 10-week interventions. The vacuoles marked by arrows indicate neutral lipid staining. H&E staining of 4 μm sections. Scale bar = 20 μm. CS = control diet and sedentary; CME = control diet with ME exercise; CHI = control diet with HI exercise; HS = high-fat diet and sedentary; HME = high-fat diet with ME exercise; HHI = high-fat diet with HI exercise.

Extensive hepatocyte vacuolation, indicating the accumulation of lipid droplets, was observed in the livers of all HFD rats (Fig 3). Vacuolation was dramatically higher in HS rats compared to all other groups. Fewer lipid droplets were observed in the livers of HME and HHI rats compared with the HS rats. There was no significant difference in vacuolation when comparing the three CD groups (CS, CME, and CHI) to each other or to the HHI rats.

### 3.5. Serum glucose and lipid profiles

Table 3 shows serum metabolite information including the glucose and lipid profiles obtained from the post-interventional rats. Diet, as a main factor, significantly affected glucose and lipid profiles. Exercise intervention had a great effect on GLU, QUICKI, and TG. HI training had greater effect on GLU (HI vs sedentary, *P* < 0.01, CI: [−4.24 to −1.06]), TG (HI vs sedentary, *P* < 0.01, CI: [−0.37 to −0.05]), and QUICKI (HI vs sedentary, *P* = 0.056, CI: [–0.02 to 0.00]) compared to sedentary groups. The combination of diet and exercise had an interactive effect on GLU and QUICKI, however, no significant differences in insulin levels were found between different groups.

**Table 3:** Characteristics and comparisons of serum variables induced by different diets and exercises.

	CS	CME	CHI	HS	HME	HHI	Diet		Exercise		Diet × exercise	
	n = 11	n = 8	n = 8	n = 10	n = 8	n = 8	Main effect	η2	Main effect	η2	Interaction	η2
**GLU (mmol/L)**	7.86±1.36	7.93±1.78	7.60±1.07	13.95±0.2.23	11.06±2.47*	8.63±2.11**	F = 42.55, P<0.01	0.324	F = 9.98, P<0.01	0.152	F = 8.35, P<0.01	0.127
**Insulin (mU/L)**	13.75±2.15	12.19±0.563	13.27±1.17	15.13±1.43	14.87±1.49	14.69±1.19	F = 17.35, P<0.01	0.253	F = 1.52, P>0.05	0.044	F = 0.93, P>0.05	0.027
**QUICKI**	0.305±0.008	0.308±0.015	0.305±0.007	0.280±0.006	0.287±0.015	0.298±0.011**	F = 33.60, P<0.01	0.348	F = 3.56, P<0.05	0.074	F = 3.30, P<0.05	0.068
**TG (mmol/L)**	0.33±0.09	0.243±0.05	0.216±0.06	0.607±0.09	0.405±0.09	0.284±0.05*	F = 10.16, P<0.01	0.139	F = 6.35, P<0.01	0.173	F = 1.37, P>0.05	0.037
**TC (mmol/L)**	1.23±0.26	1.27±0.10	1.13±0.11	1.54±0.14	1.45±0.09	1.534±0.20	F = 39.66, P<0.01	0.431	F = 0.40, P>0.05	0.009	F = 1.81, P>0.05	0.039
**HDL-C (mmol/L)**	0.44±0.07	0.45±0.05	0.41±0.05	0.44±0.04	0.40±0.05	0.42±0.05	F = 0.65, P>0.05	0.013	F = 0.74, P>0.05	0.029	F = 0.97, P>0.05	0.038
**LDL-C (mmol/L)**	0.18±0.07	0.178±0.07	0.155±0.05	0.23±0.01	0.21±0.04	0.23±0.03	F = 12.98, P<0.01	0.210	F = 0.31, P>0.05	0.010	F = 0.64, P>0.05	0.021
**Corticosterone (ng/mL)**	40.84±2.13	43.79±1.05*	42.19±3.18	47.91±2.11	43.69±2.79**	41.15±1.71**	F = 10.03, P<0.01	0.090	F = 6.89, P<0.01	0.123	F = 18.35, P<0.01	0.328

*Note*: CS = control diet and sedentary; CME = control diet with ME exercise; CHI = control diet with HI exercise; HS = high-fat diet and sedentary; HME = high-fat diet with ME exercise; HHI = high-fat diet with HI exercise; n = number; Value = mean ± SD; η^2^ indicates effect size. GLU = serum glucose; QUICKI = the quantitative insulin sensitivity check index; TG = triglycerides; TC = total cholesterol; HDL-C = high-density lipoprotein cholesterol; LDL-C = low-density lipoprotein cholesterol. Statistical significance using one-way ANOVA: * vs sedentary group (*, *P* < 0.05; **, *P* < 0.01).

HHI and HME rats had a significantly lower level of GLU (F = 12.33, *P* < 0.01; HHI vs HS, *P* < 0.01, CI: [−8.15 to −2.50]; HME vs HS, *P* < 0.05, CI: [−5.71 to −0.07]), and TG (F = 3.76, *P* < 0.01; HHI vs HS, *P* < 0.01, CI: [−0.64 to −0.01]) compared to HS rats. QUICKI also was significantly higher in the HHI group than in the HS group (F = 6.07, *P* < 0.01; HHI vs HS, *P* < 0.01, CI: [0.00–0.03]). No mean differences in glucose or lipid profiles were found among CD groups.

### 3.6. Serum corticosterone levels

Mean differences and comparisons in corticosterone levels in rats after the 10-week intervention are shown in Table 3. Diet, exercise and the interaction of diet and exercise had significant effects on serum corticosterone. Corticosterone levels in HI rats were lower compared with the sedentary and ME rats (HI vs sedentary, *P* < 0.01, CI: [−4.43 to −0.65]; HI vs ME, *P* < 0.05, CI: [−4.09 to 0.06]). Among the HFD groups, corticosterone level was significantly reduced in HHI (*P* < 0.01, CI: [−9.54 to −3.99]) and HME rats (*P* < 0.01, CI: [−6.99 to 1.44]) compared with HS rats. A similar difference was observed between CME and CS rats (*P* < 0.05, CI: [0.19–5.70]), however no mean difference was found between CHI and CS rats.

### 3.7. Correlations between corticosterone and metabolic variables

**Fig 4:**
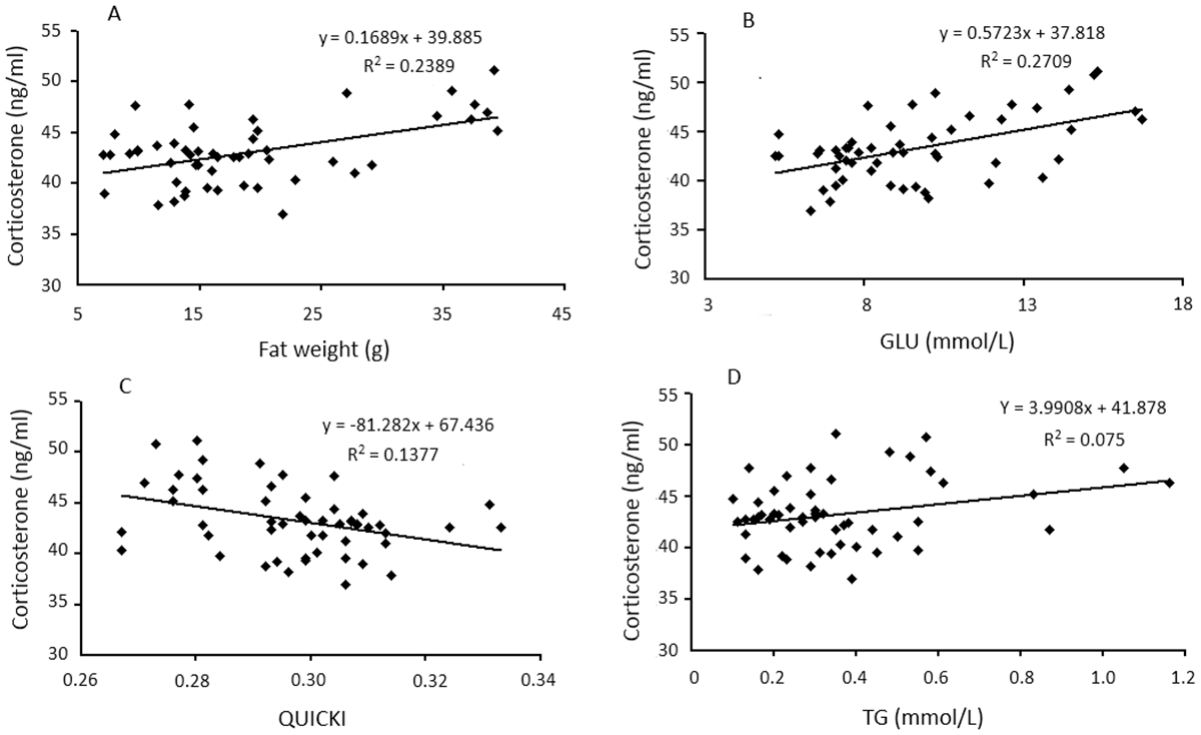
Correlations between serum corticosterone and several variables. Analyses indicated that corticosterone levels were positively associated with fat weights (A. *r* = 0.500, *r*^*2*^ = 0.239, *P* = 0.000), GLU (B. *r* = 0.520, *r*^*2*^ = 0.271, *P* = 0.000), and TG (C. *r* = 0.274, *r*^*2*^ = 0.075, *P* = 0.047), and negatively correlated with QUICKI (D. *r* = −0.375, *r*^*2*^ = 0.138, *P* = 0.006). Each diamond (♦) represents one animal (*n* = 53). CS = control diet and sedentary; CME = control diet with ME exercise; CHI = control diet with HI exercise; HS = high-fat diet and sedentary; HME = high-fat diet with ME exercise; HHI = high-fat diet with HI exercise; GLU = glucose; QUICKI = the quantitative insulin sensitivity check index; TG = triglycerides.

Corticosterone levels were positively associated with fat weights (*r* = 0.500, *P* < 0.01), GLU (*r* = 0.520, *P* < 0.01), and TG (*r* = 0.274, *P* < 0.01), whereas corticosterone levels were negatively correlated with QUICKI (*r* = −0.375, *P* < 0.01) for all groups as a whole (Fig 4).

## Discussion

Diet and physical exercise may modulate the responsiveness of the metabolic and neuroendocrine cascade, which are capable of regulating energy input and output [8, 33]. It is evident that a high-fat diet and insufficient physical activity are principal contributors for the development of chronic metabolic diseases, which are characterized by metabolic disorders and neuroendocrine disruptions [10, 13, 34]. Exercise training may counteract HFD-induced deleterious metabolic function and hypertensive responses of the HPA axis [26, 35]. The main findings of this study revealed that HFD and exercise training, independently or in combination, led to changes in metabolic phenotype and corticosterone reactions. Importantly, HI training was demonstrated to be superior to ME for improving HFD-induced weekly weight gain, metabolic disorder and corticosterone response.

In this study, the HFD with no exercise induced a disruption in metabolic phenotype, as reflected by elevated body weight, disorders in glucose and lipid profiles, and hepatic lipid accumulation. Although food intake was less in HFD compared to CD rats, total caloric consumption was greater in HFD rats. This may be explained by the high caloric density in the HFD and the efficacy of a high saturated fat diet leading to obesity. The present study showed that both ME and HI training tended to improve body composition, as well as the glucose and lipid profiles that were induced by a sedentary lifestyle and a high-fat diet. However, the form of training, as determined by the nature of the exercise stimulus, such as the intensity, duration, and activity patterns, had a profound influence on metabolic phenotype. Superior benefits of HI vs. ME training were observed for improving the HFD-induced metabolic phenotype, which led to a significant reduction in body mass, blood glucose, and hepatic lipid accumulation. Recent research found improvements in body composition, fasting glucose, and lipid accumulation after HI training intervention both in patients and rodent models with metabolic diseases [36–40]. The HI training involved intermittent bouts of physical exercise (near maximal or supramaximal effort), interspersed with periods of rest or low-intensity intervals [41]. The HI may burn more calories and induce greater adaptations over a range of physiological, performance and health-related markers when compared with matched-load traditional endurance training [41]. HI training improved beta-oxidation, which led to the intake of fatty acids in the tissue and reduced lipid accumulation in the cells [36, 42]. In addition, HI training involved a larger muscle mass, which improved glucose and fatty acid regulation during the period of exercise and recovery [43].

Notably, the beneficial effects of HI training on body and fat weight were similar to ME training in CD rats. ME training reduced fat weight and GLU, but did not improve other variables, indicating that ME training ameliorates, but did not fully normalize, some of the HFD-induced abnormal metabolic characteristics [1, 26]. This finding also suggests that both HI and ME training can decrease fat accumulation in rats fed either a CD or a high-fat diet. However, ME training did not attenuate the progression of metabolic disruption as effectively in rats fed a high-fat diet. A possible reason is that energy expenditure in the ME group was not adequate to effectively counteract the HFD-induced negative neuroendocrine response and surplus energy, which caused an abrupt increase in lipid accumulation and subsequent outcomes.

In this study, HFD-induced abnormal metabolic disruption was accompanied by an elevated circulating corticosterone level, which may indicate adrenal hyperactivity. The physiological levels of corticosterone released from the adrenal cortex plays a crucial role in regulating homeostatic function, including metabolism and fat distribution [44, 45], whereas aberrant release of corticosterone is related to the development of metabolic disorders [8, 33]. Several possible factors may explain the HFD-induced corticosterone response and its correlation with metabolic phenotype. A high-fat diet causes energetic overload that induces a continuous irregularity in HPA axis function and interference in metabolic homeostasis. When organisms fail to adjust to energy demands, it can be detrimental and may inhibit the ability to overwhelm the adaptive capacity, which can lead to the development of metabolic disturbance. Moreover, a high-fat diet may stimulate greater corticosterone secretion because of a blunted feedback mechanism, which would lead to a reduced negative feedback adjustment on the HPA axis and to an augmented corticosterone response. Additionally, a high-fat diet could reduce peripheral tissue-specific enzyme or receptor activity and sensitivity to GC [46]. Thus, it is reasonable to propose that the recruited mechanisms related to this exaggerated corticosterone response and serum metabolites are complex and may be involved in the regulation of peripheral and central homeostasis.

Our findings support the hypothesis that corticosterone release is influenced by exercise, as a main or interactive factor. It has been suggested that exercise training can counteract HFD-induced hypertensive responses of the HPA axis [26, 35]. However, few studies have determined how different exercise regimens influence or counteract the HFD-induced HPA axis activity. In the present study, both ME and HI training tended to improve corticosterone secretion induced by a sedentary lifestyle and HFD. In particular, HI training improved the corticosterone response to the greatest extent. In a previous study, a significant increase in cortisol was only found at the highest exercise intensity, when exercising at 45%, 60%, and 75% of VO_2_max [47]. The degree to which the HPA axis is activated and corresponding glucocorticoids produced may largely depend on the length and severity of the stressor. In the present study, changes in corticosterone responses, which corresponded to a stimulus responded differently and were greatly affected by the form of intervention. The HI-induced corticosterone response may be due to the enhanced sensitivity and activity of GC receptors, thus reducing corticosterone response duration [8]. Accordingly, the corticosterone responses may contribute to a positive HPA axis feedback and improved sensitivity and activity of GC receptors. It is possible that HI training triggers feedback loops, which in turn, promote the control of the HPA axis feedback and downregulate HFD-induced GC release, producing a healthy adaptive responsiveness. This would be conducive to re-stabilizing global metabolic homeostasis in a challenging environment.

Unexpectedly, the current study found that ME training tended to elevate the corticosterone level in the CD group, but it had the opposite effect in the HFD groups. This finding was inconsistent with some studies that reported increased circulating corticosterone by voluntary exercise [26, 48]. However, other research has demonstrated that wheel or treadmill running increased potentially physiological adaptations as indicated by decreased corticosterone levels [20] and improved stress resistance and HPA axis function [28, 49]. The results of this study may reflect either an independent or mixed interaction of diet and exercise, suggesting a complex mechanism that connects metabolism and the neuroendocrine system. When combined with a CD, exercise training may trigger the HPA axis releasing adequate GC to physiologically match or appropriately deal with the energy requirement response to a specific exercise. In contrast, when combined with a high-fat diet, exercise training may readjust the HFD-induced HPA axis hyperactivity, reacting with negative feedback on GC biosynthesis and secretion, thus striving to regulate the circulating corticosterone level to a physiological range. Taken together, the ME training-induced metabolic and corticosterone reactions could be the complicated interaction of diet and exercise. ME training, however, appears to be insufficient for reversing HFD-induced metabolic disorders and adrenal hyperactivity.

In the current study, a close correlation was found between corticosterone and metabolic phenotype, indicating an intrinsic mechanism between the metabolic and neuroendocrine systems. Previous studies have shown that a relationship between GC level and a high-fat diet independently induce obesity and related metabolic disturbances [16]. Alterations in central and peripheral GC production and sensitivity are common in obese humans and in rodent models of obesity, which contribute to fat accumulation in hepatic cells and increased visceral depots [33, 50] and may cause peripheral insulin resistance [16]. Organism strives to maintain GC levels within certain boundaries under special stressor stimuli, which is essential for maintaining homeostasis. Interference at any level of the HPA axis will influence other components via feedback loops and may be reflected in the metabolic phenotype and neuroendocrine response [15]. Thus, the correlation between metabolic phenotype and corticosterone observed in this study, which was stimulated by different diets and exercise, may be responsible for the dynamic but different responses to meet the physiological needs and processes for coping with the demands of homeostatic function.

### 4.1. Limitations

There were some limitations in the current study. 1) Serum metabolites and corticosterone were collected at a single and relatively late time point. This timing may not be conducive to observing the dynamically changing tendencies of those compounds. 2) No behavioral tests were done; thus, it is not known whether the HFD-induced body weight gain or the HI-induced leanness itself caused changes in corticosterone levels. 3) The interactive mechanism of diet and exercise is complex, and this study provided only partial and limited data from experimental rats. Additional mechanisms, at the molecular biological levels of signal pathways, still need to be examined. Further studies are needed to clarify the relationships between dietary type, exercise mode, and stress response.

## Conclusions

In conclusion, a high-fat diet can induce an abnormal metabolic phenotype and exacerbated release of corticosterone. Exercise training can modulate the adverse metabolic phenotype induced by a high-fat diet, though the beneficial effects depend on the characteristics of exercise training. Importantly, HI training can improve the metabolic and corticosterone responses and adaptations. Compared to ME training, HI training more effectively reduced susceptibility to HFD-induced disorders. These adaptive changes to diet and exercise may be regulated via metabolism and the neuroendocrine system. Further research is needed to elucidate the underlying mechanisms involved in HI-induced homeostatic adjustment and adaptation.
